# Relationship between the Phenylpropanoid Pathway and Dwarfism of *Paspalum seashore* Based on RNA-Seq and iTRAQ

**DOI:** 10.3390/ijms22179568

**Published:** 2021-09-03

**Authors:** Yong Zhang, Jun Liu, Jingjin Yu, Huangwei Zhang, Zhimin Yang

**Affiliations:** College of Agro-Grassland Science, Nanjing Agricultural University, Nanjing 210095, China; 2018220006@njau.edu.cn (Y.Z.); liujun825@njau.edu.cn (J.L.); jingjin_yu@126.com (J.Y.); 2020220006@stu.njau.edu.cn (H.Z.)

**Keywords:** RNA-Seq, iTRAQ, dwarfism, phenylpropanoid, *Paspalum seashore*, lignin

## Abstract

*Seashore paspalum* is a major warm-season turfgrass requiring frequent mowing. The use of dwarf cultivars with slow growth is a promising method to decrease mowing frequency. The present study was conducted to provide an in-depth understanding of the molecular mechanism of T51 dwarfing in the phenylpropane pathway and to screen the key genes related to dwarfing. For this purpose, we obtained transcriptomic information based on RNA-Seq and proteomic information based on iTRAQ for the dwarf mutant T51 of *seashore paspalum*. The combined results of transcriptomic and proteomic analysis were used to identify the differential expression pattern of genes at the translational and transcriptional levels. A total of 8311 DEGs were detected at the transcription level, of which 2540 were upregulated and 5771 were downregulated. Based on the transcripts, 2910 proteins were identified using iTRAQ, of which 392 (155 upregulated and 237 downregulated) were DEPs. The phenylpropane pathway was found to be significantly enriched at both the transcriptional and translational levels. Combined with the decrease in lignin content and the increase in flavonoid content in T51, we found that the dwarf phenotype of T51 is closely related to the abnormal synthesis of lignin and flavonoids in the phenylpropane pathway. CCR and *HCT* may be the key genes for T51 dwarf. This study provides the basis for further study on the dwarfing mechanism of *seashore paspalum*. The screening of key genes lays a foundation for further studies on the molecular mechanism of *seashore paspalum* dwarfing.

## 1. Introduction

*Seashore paspalum* (*Paspalum vaginatum Swartz*) is a perennial herb of Paspalum L., which is native to North and South America [1]. It is also one of the most widely used warm-season turfgrasses in tropical and subtropical climates [2]. Compared with other grasses, *seashore paspalum* is advantageous due to its excellent tolerance to salt, drought, waterlogging, barren land, and wear [3]. Mowing, as the most basic cultural practice applied to turf, is labor-intensive and time-consuming. Several plant growth regulators, such as ethephon, trinexapac-ethyl, and endothal have been developed and applied to turf to reduce or slow the growth of grasses, thus decreasing the mowing required. An alternative to decrease mowing frequency is to develop dwarf grass cultivars.

Dwarfism, an important agronomic trait, has been studied extensively in field crops and model plants such as rice (*Oryza sativa Larfism*) [4], wheat (*Triticum aestivum* L.) [5], and Arabidopsis (*Arabidopsis thaliana* L.) [6].It has been shown that most dwarfing genes regulate the balance of endogenous phytohormone(s) to disturb cell proliferation and/or cell expansion, thus resulting in dwarfism [7,8,9,10].

In addition to these phytohormone-related dwarfing genes and mechanisms, the phenylpropanoid pathway is also involved in dwarfism [11,12]. The phenylpropane pathway is a universal pathway in the secondary metabolism of plants, catalyzed by more than 10 enzymes, mainly including phenylalanine ammonialyase (PAL), cinnamate 4-hydroxylase (C4H), caffeoyl shikimate esterase (CSE), coumarate 3-hydroxylase (C3H), ferulate-5-hydroxylase (F5H), 4-coumarate CoA ligase (4CL), coumaroyl-coenzyme A-3-hydroxylase (CCH), caffeic acid 3-O-methyl transferase (COMT), cinnamoy1-CoA reductase (CCR), cinnamyl alcohol dehydrogenase (CAD), sinapyl alcohol dehydrogenase (SAD), cafeoy1-CoA3-O-methy1transferase (CCoAOMT), and hydroxycinnamoyl CoA:shikimate hydroxycinnamoyl transferase (HCT). Phenylpropanoid metabolism in plants mainly includes phenylalanine metabolism and the synthesis of secondary metabolites such as lignin and flavonoids. Studies have shown that plant dwarfing through the phenylpropane pathway usually leads to a decrease in lignin content, some of which is accompanied by an increase in flavonoid content. Knockout of *S-adenosyl-L-homocysteine hydrolase* (*SAHH*) results in dwarfism with increasing cytokinin (CK) [13,14,15]. Silencing of *CAD* in *Brachypodium distachyon* causes dwarfism with decreasing lignin content [16]. Silencing of *HCT* in Arabidopsis results in dwarfism by accumulating endogenous flavonoid [17]. Downregulation of *CCR1* in *Salvia miltiorrhiza* Bunge causes dwarfism with decreasing lignin content [18].

RNA-Seq is an essential tool for analyzing differential gene expression and gene regulatory networks at the transcriptional level. It has also been widely used in plants lacking complete genomic information to find transcriptional evidence [19,20,21]. At present, RNA-Seq has been applied to determine the molecular mechanism of dwarfing regulation in Kentucky Bluegrass [22], Brassica [23], napus pear [24], and other plants, as well as the mining of dwarfing-related genes. Proteomic analyses, providing translational insights, have also been widely used in determining proteomics of dwarfing mutants to quickly find dwarfing-related proteins [25,26,27]. Many different methods can be used to compare protein levels, among which isobaric tags for relative and absolute quantitation (iTRAQ) is receiving increasing attention. Since iTRAQ can quantitatively analyze almost any protein sample and has higher sensitivity and high quantitative accuracy, it has become an increasingly widely used quantitative proteomics technology [28]. This method uses relative and absolute isobaric quantification techniques. Compared with two-dimensional electrophoresis (2-DE), isotopic affinity labeling (ICAT), and differential gel electrophoresis (DIGE) methods, iTRAQ provides improved quantitative repeatability and higher sensitivity and is therefore more widely used in proteomics research [29]. Additionally, the results of RNA-Seq can facilitate the identification of novel and known proteins and their roles in target traits [30,31].

The scarcity of genome information poses an obstacle to developing new cultivars of seashore paspalum by modern genetic and genomic methods [32]. For non-model species such as seashore paspalum that lack the sequenced genome, RNA-Seq is a valuable tool for the development of new genetic resources. To the best of our knowledge, there are some applications of RNA-Seq technology in *seashore paspalum* [33]; however, reports on the RNA-Seq of dwarfing mutants of *seashore paspalum* are lacking, and the iTRAQ of seashore paspalum has not been reported. In this study, we combined RNA-Seq and iTRAQ to construct unique transcripts and proteomes of T51 and WT and then screened dwarfism-related genes and proteins to understand the dwarfing mechanism of T51. To the best of our knowledge, this study provides the first transcriptome and proteome profile for dwarfing mutants of *seashore paspalum*, laying the foundation for screening the genes related to turf dwarfs in the future.

## 2. Results

### 2.1. Phenotypic Characterization of T51 and WT

**Figure 1:**
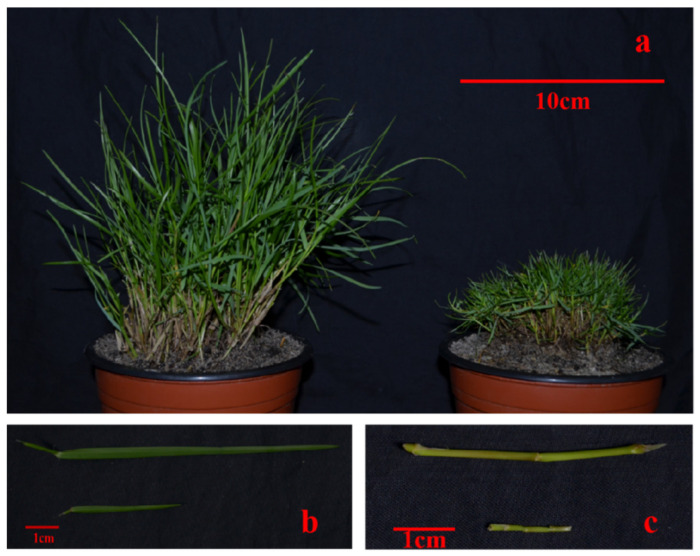
Phenotypic differences between WT and T51. (**a**) Plant types of WT and T51. (**b**) Leaves of WT and T51. (**c**) Erect stem of WT and T51.

Compared with WT, T51 showed a dwarf phenotype (Figure 1). The plant height of T51 was significantly shorter than that of WT (Table 1). In addition, the lengths of the leaves and erect stem of T51 were significantly shorter than those of WT (Table 1).

### 2.2. Transcriptional Analysis of DEGs

**Figure 2:**
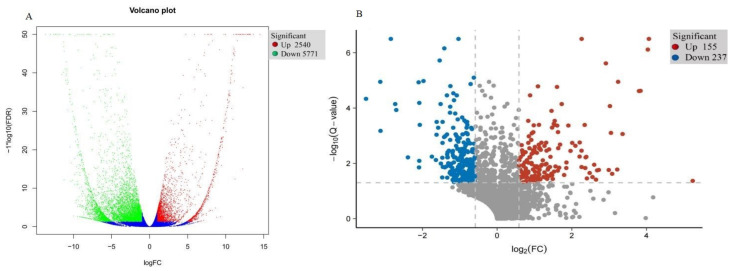
(**A**) Expression pattern of differentially expressed genes in T51 and WT. (**B**) Expression pattern of differentially expressed proteins in T51 and WT.

To verify transcriptional-level changes, two cDNA libraries (i.e., WT and T51) were constructed using the total RNA extracted from the leaves of WT and T51. After removing low-quality reads and adaptor sequences, 66.41, 64.50, 53.53, 53.51, 48.88, and 65.56 million clean reads were obtained for T51-1, T51-2, T51-3, WT-1, WT-2, and WT-3, respectively, with 76.33%, 74.88%, 75.17%, 75.31%, 74.88%, and 76.18% of the reads mapped to the reference sequence, respectively, which was a transcriptome assembled by Trinity (Table S1). Finally, 54,573 unique genes were detected in T51_WT (Table S1). The DEGs were filtered with FDR < 0.05, |log_2_ fold-change| > 1 in T51_WT for comparison. Compared with the WT group, the T51 group had 8311 (2540 upregulated and 5771 downregulated) significant DEGs (Figure 2A).

### 2.3. Identification of DEPs Using iTRAQ Technology

The DEPs in T51_WT comparison were identified and quantified using iTRAQ and LC–MS/MS analysis. Accordingly, 384,388 spectra were generated, and 23,271 unique peptides and 2910 proteins were identified with an unused score of ≥1.3 and %Cov ≥ 95 (Table S2). Among these proteins, there were 392 (155 upregulated and 237 downregulated) DAPs in T51_WT, with a fold-change of ≥1.5 (mean value of all compared groups) or ≤0.67 and a *p*-value (z-test of all comparison groups) of ≤0.05 (Figure 2B).

### 2.4. Correlation Analysis between the Transcriptome and Proteome Data

**Figure 3:**
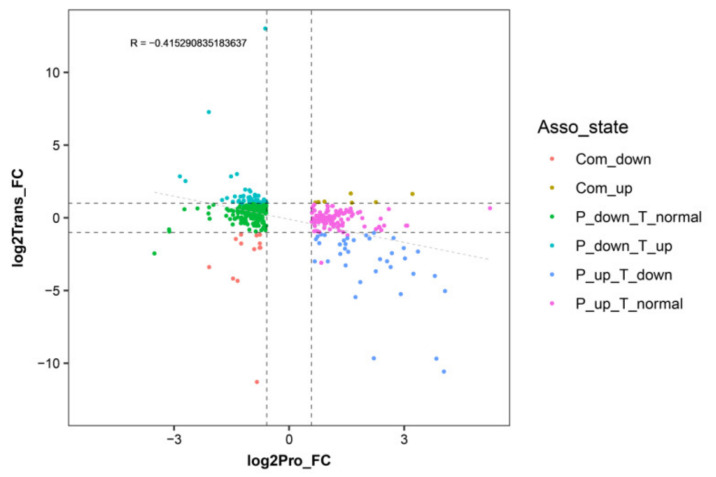
Correlation analysis between differentially expressed genes and proteins in WT and T51 samples. The *x*-axis illustrates the expression pattern of the differentially expressed proteins (DEPs), and the *y*-axis illustrates the expression pattern of their corresponding DEGs in WT and T51 samples.

An expression correlation analysis was performed between the DEPs and their corresponding DEGs (Figure 3). The detailed correlation analysis results are shown in the Supplementary Data (Table S3) Overall, most of the DEGs and DEPs showed no corresponding correlation. Among those DEGs/DEPs, 13 exhibited common upregulated expression tendency, 7 exhibited a common downregulated expression tendency, 45 exhibited downregulated expression at the proteome level and upregulated expression at the transcriptome level, 37 exhibited upregulated expression at the proteome level and downregulated expression at transcriptome level, 179 exhibited downregulation at proteome level and no difference at the transcriptome level, and 111 exhibited upregulation at the proteome level and no difference at the transcriptome level.

### 2.5. KEGG Pathway Enrichment Analysis of DEGs and DEPs

To annotate the functions of genes and proteins, we conducted a pathway enrichment analysis of the DEGs and DEPs in T51_WT based on the KEGG database. DEGs were significantly enriched in 24 pathways (Table 2), with the highest enrichment and the largest number of DEGs in ribosome and phenylpropane pathways. In addition, the DEGs also showed enrichment in plant hormone signal transduction pathways and flavonoid biosynthesis pathways.

DEPs were significantly enriched in 17 pathways (Table 3), with the highest enrichment and the largest number of DEPs in metabolic pathways and biosynthesis of secondary metabolites pathways. In addition, DEPs showed enrichment in phenylpropanoid biosynthesis pathways and flavonoid phenylalanine metabolism pathways. Comparative analysis indicated that the phenylpropanoid biosynthesis pathway is a common pathway between the transcriptome and proteome, so it was further investigated as a candidate pathway for dwarfing research.

### 2.6. Phenylpropanoid Biosynthesis Pathway

In the phenylpropanoid pathway, 91 genes and 20 proteins were found to be differentially expressed. The relationship between the DEPs and their corresponding genes is shown in Table 4, in which two DEPs are annotated as PAL, one DEP is annotated as 4CL, two DEPs are annotated as CCR, one DEP is annotated as HCT, four DEPs are annotated as UGT72E, one DEP is annotated as BRT1, one DEP is annotated as F5H, and eight DEPs are annotated as peroxidase. Among them, PAL, 4CL, CCR, HCT, and F5H are the key enzymes of lignin synthesis.

### 2.7. Validation of RNA-Seq Results

The expression levels of six genes, including TRINITY_DN 54911_c1_g2(*PAL)*, TRINITY_DN 47018_c0_g1(*4CL)*, TRINITY_DN 42134_c0_g1 (*CCR)*, TRINITY_DN 50548_c2_g2 (*peroxidase*, EC:1.11.1.7), and TRINITY_DN54341_c1_g7 (*coniferyl-alcohol glucosyltransferase*, EC:2.4.1.111), and TRINITY_DN 50277_c0_g1(*HCT*) were determined by quantitative real-time polymerase chain reaction (qRT-PCR). The expression patterns were similar to those generated from high-throughput sequencing, indicating the reliability of our transcriptome profiling data (Supplementary Materials Figure S1). The trends in the expression levels of *4CL*, *CCR*, *HCT*, and *coniferyl-alcohol glucosyltransferase* (EC:2.4.1.111) revealed by qRT-PCR were inconsistent with the changes in the abundance of their encoded proteins as detected in the iTRAQ analysis. These results suggested that deep sequencing represents an accurate and efficient method for analyzing *seashore paspalum* gene expression levels and the abundance of their corresponding proteins.

### 2.8. Difference in the Lignin and Flavone Contents in the Leaves of T51 and WT

**Figure 4:**
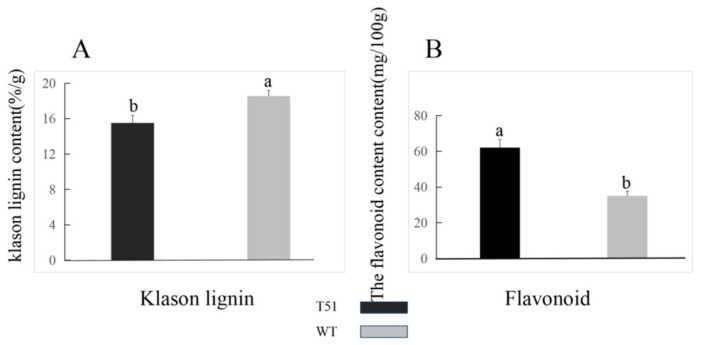
(**A**) Klason lignin contents. (**B**) Flavonoid contents. Vertical lines above the means bars indicate standard error (*n* = 3; *p* < 0.05) using Tukey’s HSD post hoc test; a,b represent significant differences between treatments.

Since two DEPs were upregulated in the flavonoid pathway (Table S4), and three DEPs were downregulated in the lignin synthesis pathway, the contents of flavonoid and lignin differed between T51 and WT. The difference in the lignin content between T51 and WT was confirmed by the content of Klason lignin, which was significantly lower in T51 than in WT (Figure 4A); the content of flavonoid in T51 was significantly higher than in WT (Figure 4B).

## 3. Discussion

Comprehensive quantitative proteomics and transcriptomics analysis showed that there are more DEGs than DEPs in T51_WT, and only a few DEGs encode DEPs. These results are similar to those reported in pomegranate [34], potato [35], and orchid [36]. The trend in the transcription level is not consistent with that of protein abundance. These results indicate that protein abundance is affected by post-translational modifications and cell splicing events, rather than changes in gene transcription [37]. Correlation analysis showed that protein abundance is negatively correlated with the expression level of the corresponding genes (Figure 3). An earlier study also reported a relatively poor correlation between transcriptome and proteomic data [38]. One possible explanation for the low correlation between the transcription level and protein abundance is that the transcription level fluctuates more quickly than that of protein translation and modification. Therefore, the change in protein abundance occurs after the level of corresponding transcripts was stable [36]. Our results suggest that iTRAQ and transcriptome analysis are complementary methods for analyzing candidate proteins involved in specific physiological processes, including dwarfing of *seashore paspalum*.

A KEGG pathway enrichment analysis was completed to avoid false positive results, and the common pathways, namely the phenylpropane pathway, shared by DEPs and DEGs were selected for further study. In plants, the phenylpropane biosynthesis pathway can be divided into the common pathway and the specific pathway.

PAL is the first enzyme in the public phenylalanine biosynthesis pathway. In many plants, *PAL* is encoded by polygene family members and plays different roles in phenylpropane metabolism [39,40]. *PAL1* and *PAL2* were proven to be related to the synthesis of lignin and flavonoids in *Arabidopsis thaliana*, so the activity of *PAL* is a key factor in the synthesis of many phenolic compounds and lignin [41,42]. Inhibition of *PAL* activity is often accompanied by abnormal growth. For example, transgenic tobacco, which inhibits *PAL* expression, shows a decrease in lignin content, and the growth and development of plants are also seriously affected [43,44]. Reducing *PAL* gene activity in Arabidopsis leads to extreme dwarfing and sterility [45]. The last key enzyme in the phenylpropanoid public metabolism pathway is 4CL, which usually exists in plants as a gene family [46]. Different plants have different *4CL* functions, but the lignin content of transgenic plants that inhibits *4CL* activity shows a downward trend. Inhibition of *4CL* activity leads to the decrease in lignin content in tobacco and Arabidopsis; transgenic Arabidopsis grew normally, but transgenic tobacco showed dwarfing [47,48]. Compared with WT, two PAL proteins and one 4CL protein in T51 were upregulated, indicating that PAL and 4CL are not the reason for the decrease in lignin content in T51.

**Figure 5:**
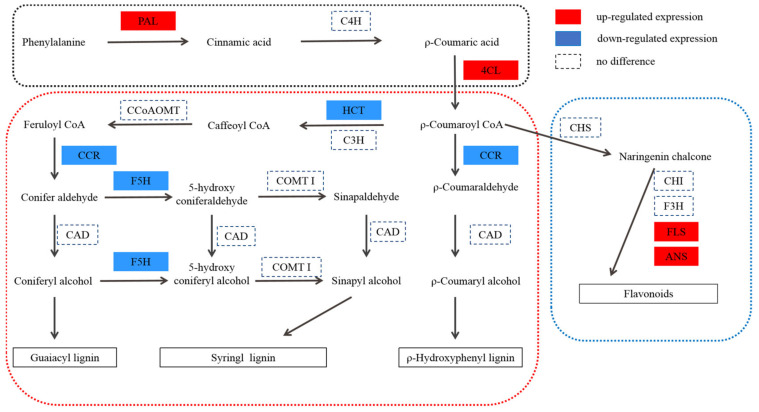
Protein expression patterns of phenylpropane pathway in T51. P-coumaroyl CoA is at the crossroads of metabolic routes leading either to flavonoids or to monolignols. *P*-coumaroyl CoA is at the crossroads of metabolic routes leading either to flavonoids or to monolignols. PAL, phenylalanine ammonia lyase; C4H, C4-hydroxylase (CYP73A5); 4CL, 4-coumaroyl-CoA ligase; CHS, chalcone synthase; CHI, chalcone isomerase; F3H, flavanone 3-hydroxylase; FLS, flavonol synthase; ANS, anthocyanidin synthase; HCT, hydroxycinnamoyl CoA:shikimate hydroxycinnamoyl transferase; C3H, C3-hydroxylase (CYP98A3); CCR, cinnamoyl-CoA reductase; CAD, cinnamyl alcohol dehydrogenase; F5H, ferulate 5-hydroxylase (CYP84A1); CCoAOMT, caffeoyl-CoA O-methyltransferase; COMT I, caffeic acid O-methyltransferase of class I.

CCR is the first key enzyme in the specific pathway of lignin synthesis. According to the current cloned plant *CCR* genes, *CCR1* is mainly related to lignin synthesis, whereas *CCR2* is mainly related to plant stress resistance [49]. Transgenic tobacco, with serious *CCR* activity downregulation, not only showed a significant decrease in lignin content (the decrease in G content resulted in the increase in the S/G ratio) but was also accompanied by abnormal growth, such as plant dwarfism [50]. The *CCR* gene of deficient Arabidopsis mutant irx4 [51] also showed a similar situation, that is, a decrease in lignin content (50% of that of the wild type) and abnormal plant growth. It can be seen that inhibition of *CCR* activity alone can reduce the biosynthesis of lignin and affect the growth and development of plants. F5H is one of the key enzymes in lignin biosynthesis, which may be the control point of S-lignin synthesis. The Arabidopsis mutant fah1, lacking *F5H* activity, has the same phenotype as the wild type, and its lignin contains only G lignin [52]. The content of s-lignin in transgenic tobacco and Arabidopsis overexpressed with F5H increased significantly, whereas g-lignin biosynthesis was inhibited significantly [53,54]. HCT is a recently studied enzyme in the biosynthetic pathway of lignin. In *Arabidopsis thaliana*, *HCT* gene silencing resulted in plant dwarfing, decreased lignin content, and composition changes [55]. Studies in *Pinus radiata* [56] and *Medicago sativa* [57] showed that changes in *HCT* gene expression levels can affect lignin synthesis and content levels in plants. The transgenic *Medicago sativa* lines with downregulation of *HCT* expression showed a phenotype with a significant decrease in lignin content, growth retardation, and biomass reduction [57]. Compared with WT, the expression levels of CCR (DEP), F5H (DEP), and HCT (DEP) were downregulated in T51, which is consistent with the lower lignin content in the leaves (Figure 4A). Lignin is a component of the plant secondary cell wall. The differential expression of lignin content and the lignin synthesis gene in T51 may lead to changes in the T51 cell wall structure. Therefore, we speculate that the changes in the expression of the above proteins change the composition and structure of lignin and then affect the plant type of T51, which is closely related to the dwarf phenotype of T51 (Figure 5).

Flavonol synthase (FLS) [58] and anthocyanidin synthase (ANS) [59] are key enzymes in flavonoid biosynthesis. Compared with WT, the expression levels of FLS and ANS (Table S4) were upregulated in T51, which caused the flavonoid content to be higher in T51 than in WT (Figure 4B). Most studies indicate that flavonoids are IAA transport inhibitors [60]. Recent studies suggest that flavonoids are involved in IAA metabolism [61]. Nitrilase (NIT) is a key enzyme in the IAA synthesis pathway [62]. N-hydroxythioamide S-beta-glucosyltransferase(UGT74B1) can regulate the dynamic balance of IAA [63]. Our results reveal that proteins related to IAA synthesis are differentially expressed in T51. The protein NIT with accession no. TRINITY_DN51107_c0_g1 showed a lower expression, whereas the protein with accession no. TRINITY_DN50720_c1_g4 showed a higher expression in T51 (Table S5). Thus, we speculate that the accumulation of flavonoids in T51 possibly inhibits the polar transport of IAA, thereby changing the IAA content and ultimately leading to the dwarf phenotype compared with the control (Figure 5).

Flavonoids and lignin are the metabolites of the phenylpropanoid pathway, and a competitive relationship exists between them. The upregulation of FLS and ANS expressions, key enzymes in flavonoids synthesis, promotes the increased flow of metabolites in the direction of flavonoids synthesis, which indirectly results in a decrease of lignin synthesis. Based on the above results, we propose a T51 dwarfing mechanism hypothesis; the abnormal synthesis of flavonoids and lignin leads to the abnormal polar transport of IAA and changes in the cell wall, finally leading to the dwarfing of T51.

## 4. Materials and Methods

### 4.1. Plant Materials

The dwarf mutant T51 of *seashore paspalum* was obtained by chemical mutagenesis using Sea Spray. After several years of field observation, the T51 dwarf phenotype was stable, so it was used in this study.

This study was conducted in June 2020 in the Nanjing Agricultural University turf grass germplasm resource garden located in Nanjing, Jiangsu Province, China. The stolons of T51 (induced by Sea Spray) and WT (Sea Spray) were planted in 11 cm diameter and 21 cm deep containers. The soil mixture was a 1:1:1 ratio of sand, soil, and perlite. *Seashore paspalum* plants were watered and fertilized as needed. T51 and WT each had 6 replicates. The mature leaves were used for the sequencing studies. Every 2 pots were used as repetitions, and 3 repetitions were collected for RNA-Seq and iTRAQ. The mature leaves were immediately frozen in liquid nitrogen for 30 min and stored at −80 °C until use.

### 4.2. Phenotypic Characterization

Plant height was considered as the natural growth height of the plant, measured with a tape measure; 10 plants were randomly selected from each treatment. Leaf length and width were measured with a Vernier caliper, and 10 plants were randomly selected from each treatment. The diameter and length of the stem were measured using a Vernier caliper, and 10 plants were randomly selected from each treatment.

### 4.3. RNA Isolation, Quantification, and Qualification

The total RNA of each sample (WT-1, WT-2, WT-3, T51-1, T51-2, and T51-3) was isolated using the RNAprep Pure Plant Kit (Tiangen Biotech, Beijing, China) according to the user manual. A Nanodrop2000 was used to detect the purity of the RNA (OD 260/280), and the quality of each RNA sample was checked on 1% agarose gel. Additionally, RNA with an RNA integrity number >8 according to the 2100 Bioanalyzer (Agilent, Palo Alto, CA, USA) was used to prepare cDNA libraries with an RNA Library Prep Kit (Illumina, San Diego, CA, USA). The resulting libraries were sequenced on a HiSeq2000 platform (Illumina), 15 November 2020.The data (raw reads) obtained from the Illumina sequencing were uploaded in the NCBI Sequence Read Archive (SRA) (accession number, PRJNA760252).

### 4.4. Gene Function Annotation

The functions of the unigenes were annotated using a series of databases, including BLASTx, against the NCBI non-redundant protein (Nr), NCBI nucleotide collection (Nt), and Swiss-Prot databases. Functional categories of putative unigenes were grouped using the Kyoto Encyclopedia of Genes and Genomes (KEGG, http://www.genome.jp/kegg/; accessed on 25 November 2020), the Clusters of Orthologous Groups of proteins (KOG/COG, http://www.ncbi.nlm.nih.gov/COG/; accessed on 29 November 2020) database, and Blast2GO against Gene Ontology (GO, http://www.geneontology.org; accessed on 4 December 2020).

### 4.5. Differential Expression Analysis

Clean reads were aligned against the assembled transcript to generate read counts using the RSEM package. The read count of each unigene was converted into FPKM values to normalize the gene expression using the FPKM method. The EdgeR package was used to analyze the differential expression of two conditions (three biological replicates per condition). Genes with an adjusted FDR < 0.05 and |log_2_ fold-change| > 1 found by EdgeR were designated as differentially expressed. Gene Ontology (GO) enrichment analysis of differentially expressed genes was performed using the GOseq R package. KOBAS software was used to find the statistical enrichment of KEGG pathways in the differentially expressed genes.

### 4.6. Protein Extraction and Protein Quantification

The sample tissue was washed with cold PBS; liquid nitrogen was fully ground, and the ground powder was transferred to a centrifuge tube. We added an appropriate amount of lysis buffer to RIPA solution containing a final concentration of 1% PMSF, which was placed in an ice bath, subjected to ultrasound for 5 min (3 s/5 s), then centrifuged at 12,000× g for 10 min at 4 °C. We then removed the supernatant, added 100% TCA solution dropwise to the final TCA concentration of 20%, and mixed it evenly. We then added an equal sample volume of CHCl3, mixed well, placed the solution on ice for 1 h, vortexing every 20 min, and then centrifuged it at 1500*× g* for 10 min. After precipitation was complete, we removed the supernatant liquid; the middle layer was the protein precipitate, and the lower layer was CHCl3 solution. We added 100 μL water and 100 μL methanol solution to the supernatant, centrifuged at 1500*× g* for 10 min, then removed the supernatant after centrifugation and adjusted the pH to 2–3 with 1 M TEAB.

We took 2 μL of protein sample, diluted it to an appropriate multiple, and determined the protein concentration by the Bradford method as follows: 2 mL of 0.02 μg/μL BSA standard was configured; BSA standards (0, 20, 40, 60, 80, 100, 120, 140, 160, 180, and 200 μL) were placed into a new 1.5 mL EP tube. We added the diluent to a final volume of 200 µL and mixed well. We placed 20 μL of the diluted BSA solution onto the microplate and set up 3 replicate wells for each concentration. We added 20 μL of the diluted protein sample to the microplate and set up 3 replicate wells for each sample. We added 180 μL of G250 color-developing solution to each well and developed the wells at room temperature in the dark for 5 min. A microplate reader was used to determine the A580 absorbance value. We calculated the concentration of the protein sample based on the standard curve and the dilution factor of the protein sample.

### 4.7. Enzyming and Desalting

We took 100 μg of protein and used U2 lysis buffer to dissolve it to about 1 μg/μL. We added 5 volumes of 100 mM TEAB and diluted the protein 6-fold. We added 1.2 μL 0.5 M CaCl_2_ and centrifuged by shaking. We then added trypsin digestion (trypsin/protein = 1:100) and incubated at 37 °C for more than 8 h. We weighed 10 mg of C18 column material, corresponding to every 100 μg of peptide sample. We activated the column material with 1 mL methanol, centrifuged with shaking, and discarded the supernatant. We added 1 mL 0.1% FA to acidify, then centrifuged and discarded the supernatant. Peptide samples were acidified with an equal volume of 0.1% FA, shaken, vortexed into a centrifuge tube, mixed quietly for 30 min, and centrifuged to discard the supernatant. The sample was then washed twice with 0.1% FA and 3% ACN for desalting and eluted with 1 mL 0.1% FA and 80% ACN. The eluted peptide was dried with a vacuum concentrator.

### 4.8. ITRAQ Labeling and Fractionation

We dissolved the peptide sample in 20 μL dissolution buffer (0.5 M TEAB), added 70 μL isopropanol, and centrifuged with shaking. Samples were labeled with an iTRAQ Reagent-8 plex Multiplex Kit (AB SCIEX, Waltham, MA, USA) according to the manufacturer’s instructions. All the labeled samples were mixed in equal amounts. Next, the labeled samples were fractionated using a high-performance liquid chromatography (HPLC) system (Thermo, Waltham, MA, USA) using a Welch C18 (5 μm, 100 A, 4.6 × 250 mm) at high pH conditions. Lastly, collected fractions were combined into 12 fractions. The combined components were desalted on a Strata-X column and dried in vacuo.

### 4.9. LC–MS/MS Analysis

Peptide samples were diluted to 1 μg/μL using on-board buffer, the sample volume was set to 8 μL, and the scanning mode was 90 min. We scanned the peptides with a mass-to-charge ratio of 350–1200 in the sample. A Triple TOF 5600 + LC/MS system (AB SCIEX, Waltham, MA, USA) was used for mass spectrometry data acquisition. The peptide samples were dissolved in 2% acetonitrile/0.1% formic acid and analyzed using a Triple TOF 5600+ mass spectrometer coupled with an Eksigent nanoLC System (AB SCIEX, Waltham, MA, USA). Peptide was loaded onto a C18 trap column (3 μm, 350 μm × 50 mm) and eluted at 300 nL/min onto a C18 analytical column (3 μm, 75 µm × 150 mm) over a 90 min gradient. The two mobile phases were buffer A (2% acetonitrile/0.1% formic acid/98% H_2_O) and buffer B (98% acetonitrile/0.1% formic acid/2% H_2_O). For information-dependent acquisition (IDA), survey scans were acquired in 250 ms, and 40 product ion scans were collected at 50 ms/scan. MS1 spectra were collected in the range of 350–1500 m/z, and MS2 spectra were collected in the range of 100–1500 m/z. Precursor ions were excluded from reselection for 15 s.

### 4.10. Proteomic Database Search

Proteinpilot 4.5 software (July 2012; ab SCIEX) was used to retrieve and analyze the mass spectrometer data. Each MS/MS spectrum was searched against a deduced protein database from RNA-Seq. The retrieval parameters were set as follows: type of search, iTRAQ 8plex (peptide labeled); enzyme, trypsin; cys alkylation, iodoacetamide; instrument, Triple TOF 5600; bias correction, TRUE; Background Correction, TRUE; ID focus, biological modifications; search effort, thorough ID; protein mass, unrestricted; detected protein threshold (unused score ≥1.3); No. distinct peptides (confidence) ≥95%. The average relative expression (fold-change ratios of differential expression between labeled protein samples), P-value, error factor, lower confidence interval, and upper confidence interval were calculated by Protein Pilot and then exported into Excel. Proteins with fold-change ratios of ≥1.5 or ≤0.67 and *p*-values of ≤0.05 were considered to be differentially expressed proteins (DEPs).

### 4.11. qRT-PCR Analysis for Validation

To validate the RNA-Seq data analysis, the total RNA used for sequencing was also used for qRT-PCR analysis for each biological replicate. Total RNA was used as the template for cDNA synthesis with reverse transcriptase (Takara Bio, Kusatsu, Japan) following the manufacturer’s instructions. qPCR and data analysis, with actin used as a reference gene to normalize gene expression, were performed as described by Wang et al. [64]. Six differentially expressed genes from RNA-Seq were validated.

### 4.12. Measurement of Klason Lignin and Flavonoid Contents

T51 and WT leaves were collected to determine the Klason lignin and flavonoid contents. Samples for measuring the Klason lignin and flavonoid contents were prepared according to the protocol of Hames et al. [65], and the contents were determined according to the protocol of Sluiter et al. [66]. Flavonoid contents were measured using the method of Quettier-Deleu et al. [67].

Phenotypic variation, correlation, and linear regression analyses were completed using SPSS (version 19.0) (IBM Corp., Armonk, NY, USA).

## 5. Conclusions

In summary, our findings suggest that the dwarfing of T51 is due to the abnormal synthesis of lignin and flavonoids in the phenylpropane pathway. Combined with the changes in the transcription and protein levels, we propose that CCR (TRINITY_DN42134_c0_g1) and HCT (TRINITY_DN50277_c0_g1) are candidate genes related to the dwarfing of the phenylpropanoid pathway in T51.

## Figures and Tables

**Table 1:** Statistical analysis of morphological differences between WT and T51.

Material	Plant Height	Leaf Length	Leaf Width	Internode Length	Diameter Width
WT	13.91a	7.74a	2.16a	1.30a	1.19a
T51	4.12b	3.29b	1.97b	0.36b	0.90b

Note: using Tukey’s HSD post hoc test. a,b represent significant difference between treatments.

**Table 2:** KEGG pathway enrichment analysis of DEGs in seashore paspalum.

Pathway ID	Pathway Name	Gene Number	*p*-Value
ko03010	Ribosome	305	1.89 × 10^−16^
ko00940	Phenylpropanoid biosynthesis	91	3.47 × 10^−11^
ko00941	Flavonoid biosynthesis	28	6.48 × 10^−6^
ko00982	Drug metabolism—cytochrome P450	33	5.60 × 10^−4^
ko01524	Platinum drug resistance	35	5.84 × 10^−4^
ko00460	Cyanoamino acid metabolism	20	1.28 × 10^−3^
ko00980	Metabolism of xenobiotics by cytochrome P450	31	1.62 × 10^−3^
ko05204	Chemical carcinogenesis	30	2.23 × 10^−3^
ko05016	Huntington’s disease	83	2.32 × 10^−3^
ko04075	Plant hormone signal transduction	58	5.12 × 10^−3^
ko05012	Parkinson’s disease	70	5.23 × 10^−3^
ko00592	alpha-Linolenic acid metabolism	24	6.98 × 10^−3^
ko05010	Alzheimer’s disease	73	1.37 × 10^−2^
ko00945	Stilbenoid, diarylheptanoid and gingerol biosynthesis	12	1.48 × 10^−2^
ko04260	Cardiac muscle contraction	18	1.52 × 10^−2^
ko04020	Calcium signaling pathway	27	1.69 × 10^−2^
ko00904	Diterpenoid biosynthesis	8	2.14 × 10^−2^
ko05134	Legionellosis	46	2.32 × 10^−2^
ko04915	Estrogen signaling pathway	32	2.68 × 10^−2^
ko00073	Cutin, suberine, and wax biosynthesis	11	3.04 × 10^−2^
ko01040	Biosynthesis of unsaturated fatty acids	21	3.05 × 10^−2^
ko00909	Sesquiterpenoid and triterpenoid biosynthesis	7	3.25 × 10^−2^
ko01220	Degradation of aromatic compounds	9	3.91 × 10^−2^
ko00944	Flavone and flavonol biosynthesis	4	3.93 × 10^−2^

**Table 3:** KEGG pathway enrichment analysis of DEPs in seashore paspalum.

Pathway ID	Pathway Name	*p*-Value	Protein Number	Up Number	Down Number
ko01100	Metabolic pathways	3.10 × 10^−6^	135	51	84
ko01110	Biosynthesis of secondary metabolites	1.80 × 10^−4^	84	37	47
ko00906	Carotenoid biosynthesis	7.70 × 10^−4^	8	1	7
ko00360	Phenylalanine metabolism	1.55 × 10^−3^	15	9	6
ko00910	Nitrogen metabolism	2.79 × 10^−3^	8	4	4
ko00940	Phenylpropanoid biosynthesis	4.44 × 10^−3^	20	14	6
ko00950	Isoquinoline alkaloid biosynthesis	5.97 × 10^−3^	5	2	3
ko00196	Photosynthesis—antenna proteins	6.49 × 10^−3^	6	0	6
ko01120	Microbial metabolism in diverse environments	8.48 × 10^−3^	45	16	29
ko00710	Carbon fixation in photosynthetic organisms	1.64 × 10^−2^	15	3	12
ko00740	Riboflavin metabolism	1.95 × 10^−2^	3	2	1
ko00960	Tropane, piperidine, and pyridine alkaloid biosynthesis	2.17 × 10^−2^	5	2	3
ko00250	Alanine, aspartate, and glutamate metabolism	2.54 × 10^−2^	9	2	7
ko04146	Peroxisome	2.88 × 10^−2^	10	7	3
ko00590	Arachidonic acid metabolism	3.51 × 10^−2^	3	0	3
ko00030	Pentose phosphate pathway	3.66 × 10^−2^	9	5	4
ko00350	Tyrosine metabolism	4.17 × 10^−2^	6	3	3

**Table 4:** Differential expression proteins and their corresponding genes in phenylpropane pathway.

Protein/Gene Number	KEGG (Ko_id and Definition)	Name	Associated State
TRINITY_DN54911_c1_g2	K10775//phenylalanine ammonia-lyase (EC:4.3.1.24)	PAL	Com_up
TRINITY_DN53461_c6_g1	K10775//phenylalanine ammonia-lyase (EC:4.3.1.24)	PAL	P_up_T_normal
TRINITY_DN47018_c0_g1	K01904//4-coumarate-CoA ligase (EC:6.2.1.12)	4CL	P_up_T_down
TRINITY_DN49758_c1_g1	K09753//cinnamoyl-CoA reductase (EC:1.2.1.44)	CCR	P_up_T_normal
TRINITY_DN42134_c0_g1	K09753//cinnamoyl-CoA reductase (EC:1.2.1.44)	CCR	P_down_T_up
TRINITY_DN50277_c0_g1	K13065//shikimate O-hydroxycinnamoyltransferase (EC:2.3.1.133)	HCT	P_down_T_up
TRINITY_DN54341_c1_g7	K12356//coniferyl-alcohol glucosyltransferase (EC:2.4.1.111)	UGT72E	P_up_T_down
TRINITY_DN51643_c2_g1	K12356//coniferyl-alcohol glucosyltransferase (EC:2.4.1.111)	UGT72E	P_up_T_normal
TRINITY_DN51903_c2_g2	K12356//coniferyl-alcohol glucosyltransferase (EC:2.4.1.111)	UGT72E	P_up_T_normal
TRINITY_DN53983_c2_g4	K12356//coniferyl-alcohol glucosyltransferase (EC:2.4.1.111)	UGT72E	P_up_T_normal
TRINITY_DN53754_c2_g1	K13068//sinapate 1 -glucosyltransferase (EC:2.4.1.120)	BRT1	P_up_T_normal
TRINITY_DN51271_c1_g1	K09755//ferulate-5-hydroxylase (EC:1.14.-.-)	CYP84A, F5H	P_down_T_normal
TRINITY_DN53699_c3_g12	K00430//peroxidase (EC:1.11.1.7)	E1.11.1.7	P_up_T_down
TRINITY_DN50408_c1_g11	K00430//peroxidase (EC:1.11.1.7)	E1.11.1.7	P_up_T_normal
TRINITY_DN51250_c1_g6	K00430//peroxidase (EC:1.11.1.7)	E1.11.1.7	P_up_T_down
TRINITY_DN50972_c4_g6	K00430//peroxidase (EC:1.11.1.7)	E1.11.1.7	P_up_T_normal
TRINITY_DN53699_c3_g7	K00430//peroxidase (EC:1.11.1.7)	E1.11.1.7	P_up_T_normal
TRINITY_DN50972_c2_g1	K00430//peroxidase (EC:1.11.1.7)	E1.11.1.7	P_down_T_normal
TRINITY_DN50548_c3_g1	K00430//peroxidase (EC:1.11.1.7)	E1.11.1.7	P_down_T_normal
TRINITY_DN50548_c2_g2	K00430//peroxidase (EC:1.11.1.7)	E1.11.1.7	Com_down

## Data Availability

Not applicable.

## Supplementary Materials

Supplementary Materials can be found at www.mdpi.com/article/10.3390/ijms22179568/s1.
