# Supplementary material for: Relationship between the Phenylpropanoid Pathway and Dwarfism of Paspalum seashore Based on RNA-Seq and iTRAQ
Source: Int J Mol Sci. 2021 Sep 3;22(17):9568. doi: 10.3390/ijms22179568 (PMC8431245; doi:10.3390/ijms22179568)
Supplement: Supplementary file 1 [file ijms-22-09568-s001.zip › supplementary files/Figure S1.pdf]

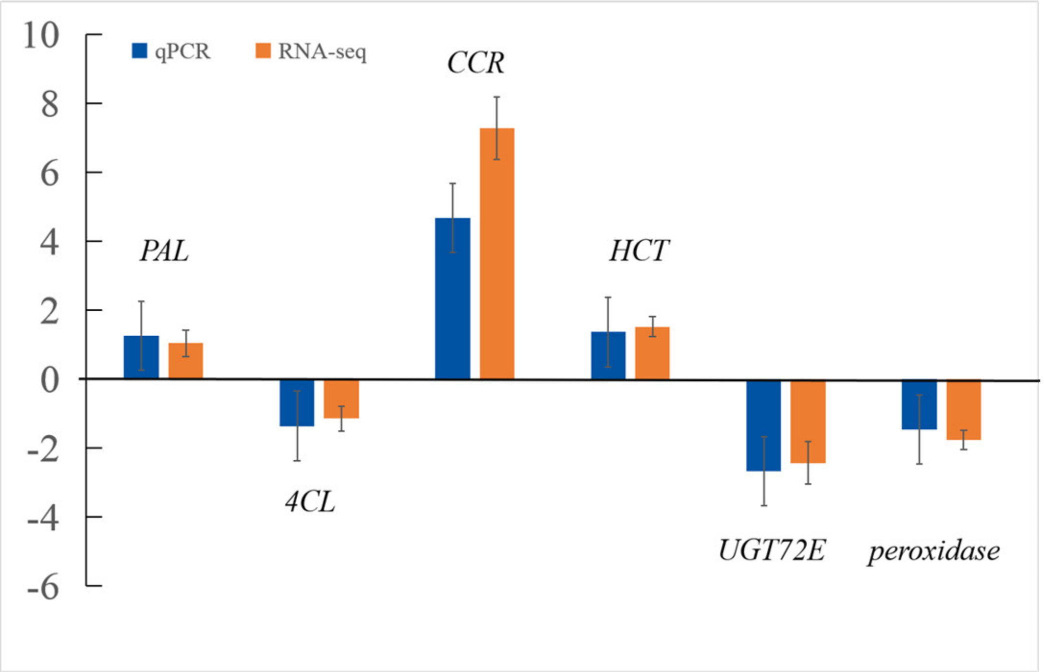

Figure S1. T51 significant difference gene qRT-PCR verification

Note: Figure S1 shows the logarithm of differential multiples of the corresponding gene, and the positive and negative values of the y axis express the gene up or down, respectively
