# Supplementary material for: Relationship between the Phenylpropanoid Pathway and Dwarfism of Paspalum seashore Based on RNA-Seq and iTRAQ
Source: Int J Mol Sci. 2021 Sep 3;22(17):9568. doi: 10.3390/ijms22179568 (PMC8431245; doi:10.3390/ijms22179568)
Supplement: Supplementary file 1 [file ijms-22-09568-s001.zip › supplementary files/Table S1.pdf]

Table S1. Transcriptome statistics

Sample-name	Total reads	Mapped reads(ratio)	Uniq mapped reads(ratio)	Multi mapped Reads(ratio)
T51-1	66409622	50689374(76.33%)	50689374(100.00%)	0.00%
T51-2	64495240	48296588(74.88%)	48296588(100.00%)	0.00%
T51-3	53532392	40240550(75.17%)	40240550(100.00%)	0.00%
WT-1	53506136	40298128(75.31%)	40298128(100.00%)	0.00%
WT-2	48881842	36605000(74.88%)	36605000(100.00%)	0.00%
WT-3	65559094	49943090(76.18%)	49943090(100.00%)	0.00%

Post assembly statistics of transcriptome data

Type	Value
Total number	54573
Total length	76398729
N50 length	2372
Mean length	1399.936397
GC%	49.45%
