# Supplementary material for: Relationship between the Phenylpropanoid Pathway and Dwarfism of Paspalum seashore Based on RNA-Seq and iTRAQ
Source: Int J Mol Sci. 2021 Sep 3;22(17):9568. doi: 10.3390/ijms22179568 (PMC8431245; doi:10.3390/ijms22179568)
Supplement: Supplementary file 1 [file ijms-22-09568-s001.zip › supplementary files/Table S2.pdf]

Table S2. Summary of protein identification information

Run Name	PSMs	Id PSMs	Peptides	Proteins
ALL	384388	104318	23271	2910

Note：ALL: Represents the sum of all sample identifications；PSMs：Total spectrum number；Id PSMs：Number of identification spectra；Peptides/Proteins：The number of identified peptides / proteins.
