# Supplementary material for: Relationship between the Phenylpropanoid Pathway and Dwarfism of Paspalum seashore Based on RNA-Seq and iTRAQ
Source: Int J Mol Sci. 2021 Sep 3;22(17):9568. doi: 10.3390/ijms22179568 (PMC8431245; doi:10.3390/ijms22179568)
Supplement: Supplementary file 1 [file ijms-22-09568-s001.zip › supplementary files/Table S3.pdf]

Table S3. Statistical relationship between transcriptome and protein expression

Compare&Class	T51:WT
Com_down	13
Com_up	7
P_down,T_normal	179
P_down,T_up	45
P_up,T_down	37
P_up,T_normal	111
