# Supplementary material for: Relationship between the Phenylpropanoid Pathway and Dwarfism of Paspalum seashore Based on RNA-Seq and iTRAQ
Source: Int J Mol Sci. 2021 Sep 3;22(17):9568. doi: 10.3390/ijms22179568 (PMC8431245; doi:10.3390/ijms22179568)
Supplement: Supplementary file 1 [file ijms-22-09568-s001.zip › supplementary files/Table S4.pdf]

Table S4. Flavonoid biosynthesis related proteins and their corresponding genes

Protein / gene number	KEGG((ko_id and definition))	name	Associated state
TRINITY_DN52624_c0_g2	K05278//flavonol synthase [EC:1.14.20.6]	FLS	P_up_T_normal
TRINITY_DN53890_c1_g2	K05277//anthocyanidin synthase [EC:1.14.20.4]	ANS	P_up_T_normal
