# Supplementary material for: Relationship between the Phenylpropanoid Pathway and Dwarfism of Paspalum seashore Based on RNA-Seq and iTRAQ
Source: Int J Mol Sci. 2021 Sep 3;22(17):9568. doi: 10.3390/ijms22179568 (PMC8431245; doi:10.3390/ijms22179568)
Supplement: Supplementary file 1 [file ijms-22-09568-s001.zip › supplementary files/Table S5.pdf]

Table S5. IAA metabolism related proteins and their corresponding genes

Protein / gene number	KEGG((ko_id and definition))	name	Associated state
TRINITY_DN50720_c1_g4	K11820//N-hydroxythioamide S-beta-glucosyltransferase	UGT74B1	P_up_T_normal
TRINITY_DN51107_c0_g1	K01501//nitrilase [EC:3.5.5.1]	E3.5.5.1	P_down_T_normal
